# Evaluation of the nasal microbiota in slaughter-age pigs and the impact on nasal methicillin-resistant *Staphylococcus aureus* (MRSA) carriage

**DOI:** 10.1186/1746-6148-10-69

**Published:** 2014-03-15

**Authors:** J Scott Weese, Mackenzie Slifierz, Mohammad Jalali, Robert Friendship

**Affiliations:** 1 Department of Pathobiology and Centre for Public Health and Zoonoses, Ontario Veterinary College, University of Guelph, Guelph, Ontario N1G2W1, Canada; 2 Department of Population Medicine, Ontario Veterinary College, University of Guelph, Guelph, Ontario N1G2W1, Canada

**Keywords:** Microbiota, Antimicrobial resistance, Staphylococci

## Abstract

**Background:**

The nasal microbiota of pigs has been poorly assessed but could play a role in carriage of important microorganisms such as methicillin-resistant *Staphylococcus aureus* (MRSA). The objectives of this study were to describe the nasal microbiota in slaughter age pigs, to evaluate the impact of farm management on the nasal microbiota and to provide a preliminary assessment of the influence of the microbiota on MRSA carriage.

**Results:**

Nasal swabs were collected from five MRSA positive and eight MRSA negative pigs on one farm that used a liquid feeding system and routine tylosin treatment, and seven MRSA negative pigs from an antibiotic-free farm that used conventional feeding. A total of 946310 sequences passed all quality control filters. The number of sequences per sample ranged from 4307 to 165656 (mean 56092, SD 40007). CatchAll analysis of richness predicted a mean of 1749 OTUs (range 213–3736, SD 996). Overall, 6291 OTUs were identified, yet 5125 (81%) were identified less than 10 times and the 12 most abundant OTUs accounted for 80.7% of sequences. Proteobacteria predominated in all but two samples. Liquid-fed/tylosin-exposed pigs had significantly lower relative abundances of Verrucomicrobia (P = 0.004), Fibrobacteres (*P* = <0.0001) and sequences unclassified at the phylum level (*P* = 0.028). When comparing only liquid-fed pigs, MRSA carriers had significantly more Bacteroidetes (*P* = 0.037) than MRSA negative pigs. 124 genera were identified, with *Moraxella* accounting for 35.4% of sequences. In the Jaccard index tree, five of eight MRSA positive pigs clustered closely together, as did six of the seven conventionally-fed pigs. A significant difference was identified between conventional and liquid-fed pigs using parsimony test with the Jaccard (P < 0.001) but not the Yue&Clayton (*P* = 0.26) index. There were no significant differences between MRSA positive and negative pigs (*P* = 0.133 and 0.175). OTUs belonging to Firmicutes were the main indicators of MRSA negative pigs, including *Lactobacillus* and another Lactobacillaceae and *Staphylococcus*.

**Conclusions:**

Farm management can influence the nasal microbiota in pigs, but no impact of the microbiota on MRSA carriage was identified. Studies that further define the impact of management on the microbiota, and the impact of the microbiota on pathogen carriage are indicated.

## Background

The body contains numerous different ecological niches that support abundant and diverse populations of microorganisms. The microbial composition of a body site (the microbiota) and the sum of its genetic materials (the microbiome) are of increasing interest since it is clear that they can play critical roles in health and disease. Recent advances in next generation sequencing and bioinformatics have resulted in the ability to characterize complex microbial populations at a level that was impossible only a few years earlier. These studies are providing remarkable insight in to the body’s microbial populations and their interaction with the host.

Most microbiota studies have focused on the intestinal tract or feces, but various other body sites harbour important microbial populations, including the nasal passages. There has been limited investigation of the nasal microbiota in pigs, with culture-based studies focusing on selected pathogens [1]. There has been some study of the microbiota of tonsillar tissue. A study of 18–20 week old pigs, bacteria from the Proteobacteria phylum predominated, with Firmicutes and Fusobacteria (phyla) being relatively common and 14 other phyla being present but rare [2]. It is likely that the tonsillar and nasal microbiotas are similar, but this has not been investigated.

While there has been limited study, the nasal microbiota may be of relevance for many reasons, including understanding of the ecology of important pig and zoonotic pathogens. One area of potential importance is methicillin-resistant *Staphylococcus aureus* (MRSA) colonization. Since 2005 [3], MRSA has become an important concern in pigs. While rarely a cause of disease in pigs, MRSA is a human health concern. High rates of MRSA carriage have been reported in pigs internationally [4-6] and pig contact is a leading source of MRSA exposure in people in some countries [7]. While MRSA carriage rates can be very high on farms, not all pigs that are exposed to MRSA become colonized. Reasons for this are unclear but one possibility is a protective effect of the endogenous nasal microbiota. If true, this could represent a potential MRSA control intervention by modification of the nasal microbiota to reduce the likelihood of MRSA carriage.

The objectives of this study were to describe the nasal microbiota in slaughter age pigs, to evaluate the impact of diet on the nasal microbiota and to provide a preliminary assessment of the potential influence of the microbiota on MRSA carriage.

## Results

Initial screening of 100 pigs from two farms resulted only in identification of farms where all or no pigs were carrying MRSA. Both MRSA positive (n = 5) and negative (n = 8) pigs were subsequently identified on a farm that used a liquid feeding system that consisted of corn, wheat shorts, soybean and whole whey. Pigs also receive tylosin until the time of slaughter. As a comparison, seven MRSA-negative pigs were enrolled from one farm that used conventional feeding practices. Pigs from this farm were not exposed to antimicrobials at any time in their lives.

A total of 946310 V4 16S RNA gene sequences passed all quality control filters. The number of sequences per sample ranged from 4307 to 165656 (mean 56092, SD 40007). CatchAll analysis of richness predicted a mean of 1749 OTUs (range 213–3736, SD 996). Significantly higher OTU richness was present in the liquid-fed group (mean 2150, SD 879) versus the conventionally-fed group (mean 1005, SD 777) (*P* = 0.0098). There was no difference between MRSA positive and MRSA negative pigs (*P* = 0.78). However, when Catchall analysis was repeated using a subsample of 4307 sequences per sample, no significant differences based on feed group or MRSA status (*P =* 0.38 and 0.69, respectively).

**Figure 1:**
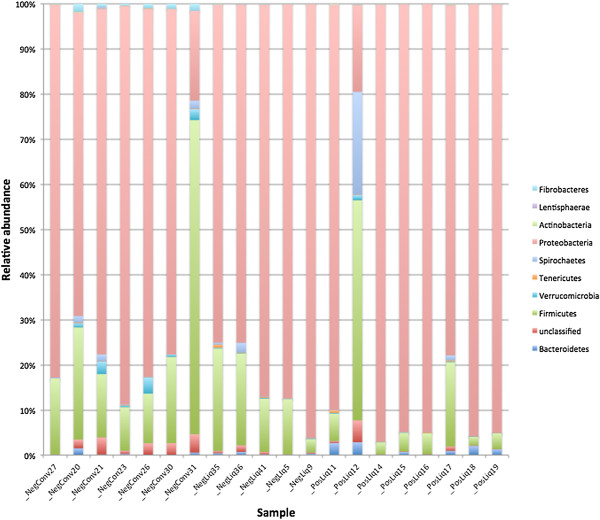
Relative abundances of bacterial phyla from nasal swabs from twenty pigs.

Overall, 6291 OTUs were identified, yet 5125 (81%) were identified less than 10 times and the 12 most abundant OTUs accounted for 80.7% of sequences. Nine different phyla were identified; however, only three (Proteobacteria, Firmicutes, Spirochaetes) accounted for >1% of sequences. Sequences that were unclassified at the phylum level accounted for 0.1% of sequences. The Proteobacteria phylum predominated in all but two samples, in which Firmicutes accounted for the majority of sequences. The relative abundances of predominant phyla are presented in Figure 1. Liquid-fed, tylosin-treated pigs had significantly lower relative abundances of Verrucomicrobia (P = 0.004), Fibrobacteres (*P* = <0.0001) and sequences unclassified at the phylum level (*P* = 0.028) compared to conventionally fed, antibiotic-free pigs. When comparing only liquid-fed pigs, MRSA carriers had significantly more Bacteroidetes (*P* = 0.037) than MRSA negative pigs.

Gammaproteobacteria was the most abundant class, accounting for 78% of sequences. Twenty-one other classes were identified, with Bacilli (6.4%), Clostridia (6.2%), Betaproteobacteria (5.1%) and Spirochaetes (1.1%) accounting for at least 1% of sequences each. At the class level, liquid-fed pigs had significantly fewer Fibrobacteria (*P* = 0.0078). There were no differences between MRSA positive and MRSA negative pigs.

One hundred twenty four different genera were identified, but *Moraxella* dominated, accounting for 35.4% of sequences (Table 1). The next three most abundant genera were also Proteobacteria of the families Moraxellaceae, *Psychrobacter* (21.2%), *Pseudomonas* (14.9%) and *Acinetobacter* (4.8%). *Mycoplasma* was found in 18 (90%) of samples but at an overall relative abundance of only 0.1%. Genera that were present at significantly different relatively abundances between liquid- and conventionally-fed pigs are presented in Table 2 An unclassified Burkholderiales (P = 0.02) and *Comamonas* (P = 0.047) were more abundant in MRSA positive pigs compared to MRSA negative pigs.

**Table 1:** Predominant genera isolated from the nasal passages of twenty healthy pigs

**Genus**	**Relative abundance**
*Moraxella*	35.4%
*Psychrobacter*	21.1%
*Pseudomonas*	14.9%
*Acinetobacter*	4.8%
*Janthinobacterium*	3.8%
*Clostridium* sensu stricto	2.4%
*Lactobacillus*	2.0%
*Aerococcus*	1.8%
*Treponema*	1.0%
Unclassified Ruminococcaceae	0.9%
*Kingella*	0.9%

**Table 2:** Genera that were significantly different (P < 0.05) in relative abundance between pigs being fed a liquid diet (n = 13) and those fed a conventional diet (n = 7)

**Genus**	**Conventional feed**	**Liquid feed**	***P *** **value**
Unclassified Porphyromonodaceae	0.023	0.007	0.04
*Macrococcus*	0.000	0.001	0.039
5 genus incertae sedis	0.014	0.0017	0.049
*Pseudomonas*	0.012	0.245	0.008
*Enhydrobacter*	0.000	0.0002	0.047
Unclassified Enterobacteriaceae	0.0047	0.0005	0.012
*Comomonas*	0.000	0.001	0.048
*Phenylobacterium*	0.00089	0.00028	0.049
*Corynebacterium*	0.000	0.00025	0.023
*Fibrobacter*	0.0093	0.00027	0.008

While *Staphylococcus* sequences were identified in every sample, they were uncommon, ranging from <0.01-8.6% (mean 0.54%, SD 2.2%). The relative abundance of *Staphylococcus* was higher in the conventionally fed pigs compared to liquid-fed, tylosin-exposed MRSA shedders (*P* = 0.02) but there was no difference overall between liquid- and conventionally fed pigs (*P* = 0.96) or MRSA positive vs MRSA negative liquid-fed pigs (*P* = 0.52).

Fifteen different genera belonging to the Enterobacteriaceae family were identified (*Escherichia, Pantoea, Serratia, Yersinia, Enterobacter, Buttiauxella, Citrobacter, Hafnia, Morganella, Obesumbacterium, Proteus, Providencia, Rahnella, Salmonella* and an unclassified genus), but only at abundances ranging up to 0.25%. *Salmonella* was identified in 4/7 (57%) pigs from the conventionally fed farm but none from the liquid fed farm.

**Figure 2:**
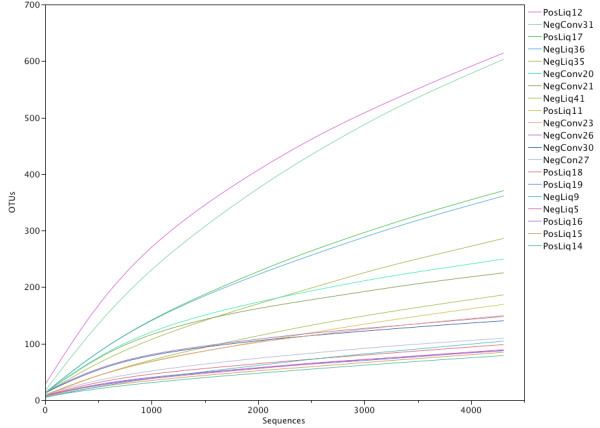
**Rarefaction curves from a subsampled population (n = 4307) of V4 16S rRNA gene sequences from porcine nasal swabs.** (Legend: Conv = conventionally fed pigs, NegLiq = liquid fed pigs not carrying MRSA, PosLiq = liquid fed pigs carrying MRSA).

Further analysis was performed on random subsampling of 4307 sequences per sample. Rarefaction curves are presented in Figure 2. Overall, excellent sample coverage was obtained with this subsampled population, as demonstrated by a mean Good’s coverage value of 0.974 (SD 0.02, range 0.92-0.99). There were no differences in coverage between groups (all *P* > 0.80). Inverse Simpson index ranged from 1.49-40.9 (mean 5.8, SD 8.4), with no differences between groups (all *P* > 0.41).

**Figure 3:**
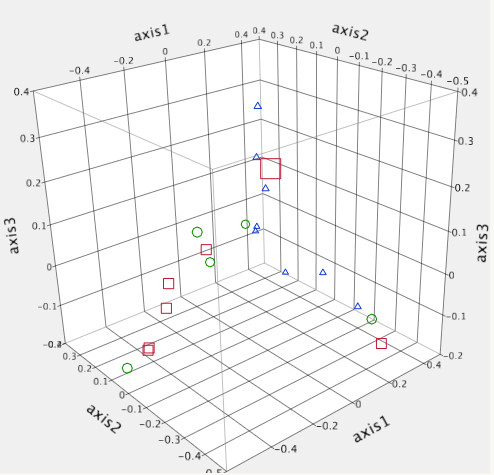
**Three dimensional principle component analysis of the nasal microbiota of twenty healthy pigs.** (Red square = conventionally fed pigs, Blue triangle = MRSA negative liquid fed pigs, Green circle = MRSA positive liquid fed pigs).

**Figure 4:**
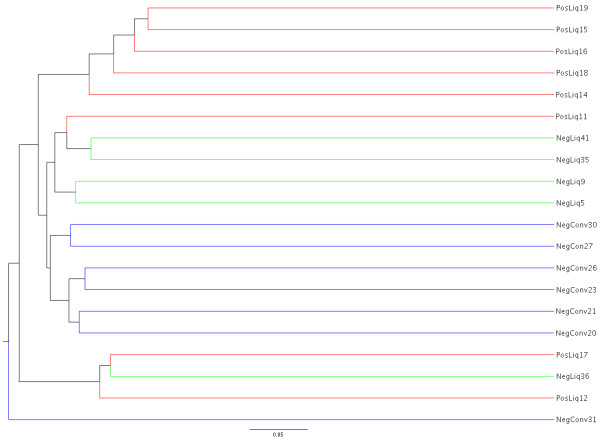
**Dendrogram of the community membership of the nasal microbiota from twenty healthy pigs, based on the Jaccard index.** (Legend: Conv = conventionally fed pigs, NegLiq = liquid fed pigs not carrying, PosLiq = liquid fed pigs carrying MRSA).

**Figure 5:**
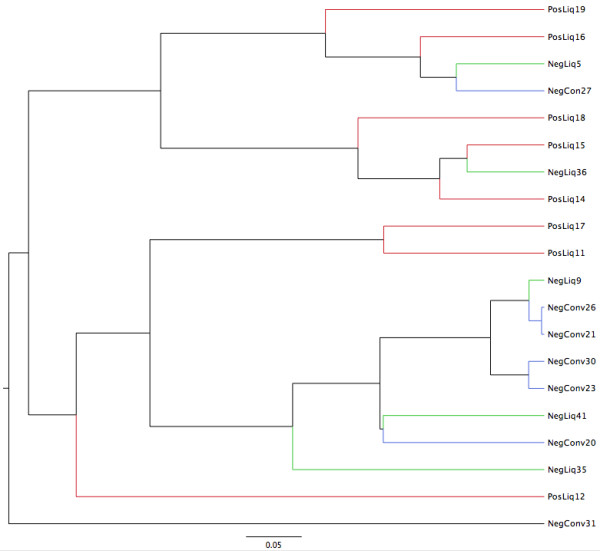
**Dendrogram of the community structure of the nasal microbiota of twenty healthy pigs based on the Yue and Clayton index of dissimilarity.** (Legend: Conv = conventionally fed pigs, NegLiq = liquid fed pigs not carrying MRSA, PosLiq = liquid fed pigs carrying MRSA).

Community structure differences can be visualized by PCA in Figure 3, as well as by dendrograms of community membership (Jaccard index) and community structure (Yue&Clayton index) (Figures 4 and 5). In the Jaccard index tree, five of eight MRSA positive pigs clustered closely together, as did six of the seven conventionally-fed pigs. However, this was not as evident with the Yue&Clayton index, which also assesses relative abundance.

A significant difference was identified between conventional and liquid-fed pigs using parsimony test with the Jaccard tree (P < 0.001) but not the Yue & Clayton tree (*P* = 0.26). There were no significant differences between MRSA positive and negative pigs (*P* = 0.133 and 0.175). No significant differences were identified by unweighted unifrac between farms or MRSA positive and negative pigs (all *P* > 0.44).

When comparing liquid-fed to conventionally-fed pigs, 20 significant (P < 0.05) indicator OTUs were identified (Table 3). Eleven significant indicator OTUs were identified when comparing MRSA positive and MRSA negative pigs (Table 4).

**Table 3:** Indicator operational taxon units (OTUs) and their relative abundance (%) for liquid-fed (n = 13) or conventionally-fed (n = 7) pigs

**Group**	**Indicator (Phylum/Class/Order/Family/Genus)**
Liquid	Proteobacteria/Gammaproteobacteria/Pseudomonales/Moraxellaceae/*Acinetobacter* (3.8%)
	Firmicutes/Bacilli/Lactobacillales/Leuconostoaceae/*Leuconostoc* (0.44%)
	Firmicutes/Bacilli/Lactobacillales/Lactobacillaceae/*Lactobacillus* (2 OTUs) (0.33%)
	Firmicutes/Bacilli/Lactobacillales/Aerococcaceae/*Aerococcus* (0.3%)
	Proteobacteria/Gammaproteobacteria/Pseudomonadales/Pseudomonadaceae/*Pseudomonas* (11.7%)
	Proteobacteria/Betaproteobacteria/Burkholderiales/Oxalobacteraceae/*Janthinobacterium* (3.8%)
	Spirochaetes/Spirochaetes/Spirochaetales/Spirochaetaceae/*Treponema* (0.11%)
Conventional	Proteobacteria/Gammaproteobacteria/Pseudomonales/Moraxellaceae/*Moraxella* (4 OTUs) (0.019%)
	Firmicutes/Bacilli/Lactobacillales/Lactobacillaceae/*Lactobacillus* (0.077%)
	Proteobacteria/Gammaproteobacteria/Pseudomonadales/Pseudomonadaceae/*Pseudomonas* (0.027%)
	Proteobacteria/Unclassified (2 OTUs) (0.046%)
	Firmicutes/Bacilli/Bacillales/Staphylococcaceae/*Staphylococcus* (0.28%)
	Firmicutes/Bacilli/Bacillales/Staphylococcaceae/Unclassified (0.009%)
	Firmicutes/Clostridia/Clostridiales/Ruminococcaceae/Unclassified (0.06%)
	Firmicutes/Unclassified (0.0035%)

**Table 4:** **Indicator operational taxon units (OTUs) for the nasal microbiota of liquid-fed pigs carrying (n = 7) or not carrying (n = 5) methicillin-resistant *Staphylococcus aureus***

**Group**	**Indicator OTU (Phylum/Class/Order/Family/Genus)**
MRSA positive	Proteobacteria/Betaproteobacteria/Burkholderiales/Oxalobacteraceae/*Janthinobacterium*
	Proteobacteria/Betaproteobacteria/Burkholderiales/Comamonadaceae/Unclassified (2 OTUs)
	Proteobacteria/Betaproteobacteria/Burkholderia/Comamonadaceae/*Comamonas*
	Bacteroidetes/Sphingobacterium/Sphingobacteriales/Sphingobacteriaceae/*Sphingobacterium*
	Proteobacteria/Betaproteobacteria/Burkholderiales/Unclassified
	Firmicutes/Bacilli/Bacillales/Staphylococcaceae/*Macrococcus*
MRSA negative	Firmicutes/Bacilli/Lactobacillales/Lactobacillaceae/*Lactobacillus*
	Firmicutes/Bacilli/Bacillales/Staphylococcaceae/*Staphylococcus*
	Firmicutes/Bacilli/Lactobacillales/Lactobacillaceae/Unclassified
	Proteobacteria/Btaproteobacteria/Neisseriales/Nesseriaceae/*Neisseria*

No OTUs were present in all samples at an abundance of 1% of greater. One OTU, belonging to *Moraxella,* was found at this minimum relative abundance in 17/20 (85%) of sample. This OTU accounted for 20% of sequences overall. Only 3 OTUs, two *Moraxella* and a *Psychrobacter*, were identified at a relative abundance of >1% for 50% of samples.

## Discussion

This study has identified remarkable microbial richness and diversity in the porcine nasal cavity. The estimated richness (1749 species) surpasses any previous porcine study but is consistent with a human study that reported an estimate of 2264 species in the anterior nares [8]. A study of the porcine tonsil reported a smaller number of OTUs (57-730/pig) [2], but that was based on analysis of a smaller number of sequences per sample compared to the current study.

Over 6000 different OTUs were identified, yet most were rare, with 12 OTUs accounting for greater than 80% of total sequences. This shows that while the population species richness may be high, evenness is low and a few species dominate. As is common in many microbial populations, a limited number of phyla accounted for >1% of sequences. Of the three main phyla in this population, Proteobacteria were dominant, similar to studies of the human nasal microbiota and the pig soft palate tonsil [2,8]. Proteobacteria includes a range of Gram negative organisms, including *Moraxella* (the dominant genus here), *Actinobacillus* and *Pasteurella.* While the predominance of Proteobacteria is consistent with a study of the pig tonsil, there were marked differences within Proteobacteria, as *Actinobacillus* and *Pasteurella* predominated in the study of porcine tonsillar tissue [2]. This could be the result of differences between the nasal passages and tonsil, as well as other factors such as pig age, farm management and laboratory methods.

Various differences were identified between pigs on the two farms. They differed in both their diet (liquid vs conventional) and antimicrobial exposure (tylosin vs no antimicrobials). Changes in the nasal microbiota could result from direct effects of the liquid feed (e.g. inoculation of microorganisms from feed into the nasal passages while eating) or indirect effects through modification of the fecal microbiota and altered environmental bacterial exposures. The impact of liquid whey feeding on the fecal microbiota was assessed in mature pigs [9]; but it was a cloning-based study that involved very small numbers of clones and provides little insight into the results of this study. Reasons for the significant lower relative abundance of Verrucomicrobia and Fibrobacteres is not clear. A decrease in Fibrobacteres is more understandable since this is a phylum of cellulose-degrading bacteria that would presumably be of less use in the intestinal tract of pigs fed liquid feeds. This difference may therefore indicate a change in the gut microbiota that is subsequently reflected in the nasal microbiota from environmental exposure. The other potential influence on the nasal microbiota is antimicrobial exposure of the liquid-fed group. Local or systemic effects could potentially modify the nasal microbiota, or could modify the fecal microbiota and indirectly impact the nasal microbiota.

Conflicting results were obtained for different tests of community structure and membership. A difference between feeding types was noted using the parsimony test and the Jaccard index. This can be visualized by assessment of the Jaccard tree, which also shows clustering of the MRSA-positive pigs. Yet, there was no significant difference in community structure based on analysis of the Yue and Clayton tree, which includes relative abundance, and no difference with Unifrac or AMOVA. The relevance of conflicting results such as these is hard to interpret and could relate to differences in the statistical measures. Specifically, differences in rare OTUs with relative stability of abundant OTUs would have more of an impact of the Jaccard index (which only assesses community membership, not relative abundance). Limitations in statistical power must also be considered. While there was not agreement between all tests and there were many similarities between groups, some differences between pigs that were, or were not, carrying MRSA were noted, as were differences between pigs on different diets. These results indicate that further study of these differences to better characterize them and try to discern cause vs effect are indicated. The relative impact of diet and antimicrobial exposure cannot be discerned with this study design, and studies targeting specific management aspects are required.

There was a limited core microbiota, with no OTUs found in all samples and only one (accounting for 20% of sequences) found in 85% of samples. This is somewhat similar to humans, where only 2 OTUs, accounting for 17% of sequences, were identified in the anterior nares [8].

Indicator analysis is an ecological tool that is used to identify species that define an environment. By evaluating species fidelity (relative abundance of the species in the population) and exclusivity, species-habitat associations can be identified beyond simply comparing relative abundances. Numerous indicator OTUs were identified here, indicating bacterial groups that deserve further study to clarify their association with diet or MRSA status and reasons for that. Interestingly, OTUs from Firmicutes were the main indicators of MRSA negative pigs, including *Lactobacillus* and another Lactobacillaceae and, perhaps surprisingly, *Staphylococcus*. Whether these might be protective for MRSA colonization cannot be stated with these data but these results raise interesting questions since re-population of the nasal microbiota with these species (e.g. through feed) could represent a practical intervention to reduce MRSA carriage. Further study of staphylococci, perhaps by using PCR targets with greater staphylococcal species resolution (e.g. *sodA*) might provide more insight into this potential protective effect. These results also show that evaluation of indicator species may provide more insight than simply comparing relative abundances between groups.

Numerous genera were over- or under-represented between MRSA positive and negative, or conventionally- or liquid-fed pigs. These are too numerous to discuss, but they, along with indicator species, provide the basis for consideration and further study of individual components of the microbiota that might be influenced by diet and/or influence MRSA status. Many other genera had P values between 0.05 and 0.1 (data not presented), indicating that larger study might identify more organisms of interest. At the same time, though, the number of statistical comparison that were performed needs to be considered, as some statistically significant differences could be generated by random chance with such a large number of comparisons. Nonetheless, these data can form the starting point for further studies.

Within-farm matching was desired because of the unknown potential for inter-farm variation. A complicating factor in this study was the difficulty in identifying farms where both positive and negative pigs of the target age were present, since an ‘all-or-nothing’ effect was apparent on many farms. This, along with poor DNA yield from some nasal swabs, ultimately limited the number of samples available for analysis. The small sample size must be considered and study of larger populations and different farms would be useful. The presence of two apparent outliers, with large proportions of Firmicutes sequences, may have further weakened statistical power. Nonetheless, this study provides useful information about the nasal microbiota in pigs and raises some points for further study of both the impact of diet on the nasal microbiota and the role of the nasal microbiota in MRSA carriage.

Given the behaviour of pigs and their environment, it is likely that the nasal microbiota consists of resident nasal commensals and transient environmental contaminants. That likely explains the finding of enteric organisms like *Salmonella,* yet those were of very low abundances and the predominant components of the nasal microbiota were Proteobacteria that are uncommon in the intestinal tract (and therefore presumably the barn environment). Fecal contamination of the nasal passages might explain the two outliers that had a large number of Firmicutes, given the predominance of this phylum in porcine feces [10].

## Conclusion

The nasal passages of slaughter-age pigs harbour a very rich and diverse microbial population that can apparently be influenced by diet and/or farm management practices. While the impact of the microbiota on MRSA carriage is unclear, these data suggest that further study of the role of the nasal microbiota on MRSA carriage and the potential that the nasal microbiota could be specifically manipulated by diet or antimicrobials warrant further study.

## Methods

### Study population and sample collection

Pigs from four commercial swine operations on southern Ontario, Canada were enrolled. Pigs were sampled 1–2 weeks prior to slaughter and clinically normal. Nasal swabs were collected using rayon-tipped culture swabs. Paired swabs were collected. One swab was tested for the presence of MRSA through selective enrichment culture, as has been previously described [4]. The other was placed in a sterile glass tube and stored at -80°C until MRSA culture results were obtained. The goal was to obtain farm-matched groups of MRSA-positive and MRSA-negative pigs for microbiota analysis. The study was approved by the University of Guelph Animal Care Committee.

### DNA extraction and quality control

DNA extraction was performed using a commercial kit (E.Z.N.A. Stool DNA Kit, Omega Bio-Tek Inc., Doraville, Georgia, USA) following the manufacturer’s “stool DNA protocol for pathogen detection”. DNA quantity and quality were accessed by spectrophotometry (NanoDrop, Roche, Mississauga, Canada).

### 16S rRNA gene amplification and sequencing

PCR amplification of the V4 region of the 16S rRNA gene was designed based on Caporaso et al. 2010 [11] using the primers forward S-D-Bact-0564-a-S-15 (5′-AYTGGGYDTAAAGNG-3′) and reverse S-D-Bact-0785-b-A-18 (5′-TACNVGGGTATCTAATCC-3′). The forward and reverse primers were designed containing an overlapping region of the forward and reverse Illumina sequencing primers (TCGTCGGCAGCGTCAGATGTGTATAAGAGACAG and GTCTCGTGGGCTCGGAGATGTGTATAAGAGACAG, respectively) in order to anneal them to primers containing the Illumina adaptors plus the 8 bp identifier indices (forward: AATGATACGGCGACCACCGAGATCTACAC-index-TCGTCGGCAGCGTC; reverse: CAAGCAGAAGACGGCATACGAGAT-index-GTCTCGTGGGCTCGG). A 50 ul reaction was performed with 25 μL of Kapa2G Fast HotStart ReadyMix 2X (KapaBiosystems); 1.3 μL of BSA (Bio-Rad); 18.9 μL of PCR-grade H_2_O, 2 μL of DNA template, and 0.4 μL of both the forward and the reverse 16S primers (10 pmol/μL). The following PCR conditions were used; 3 min at 94°C for denaturing, and 30 cycles of 45 sec at 94°C for denaturing, 60 sec at 50°C for annealing and 90 sec at 72°C for elongation followed by a final period of 10 min at 72°C and kept at 4°C until purification.

PCR products were evaluated by electrophoresis in 2% agarose gel and purified with the Agencourt AMPure XP (Beckman Coulter Inc, Mississauga, Ontario, Canada) by mixing 20 μL of amplicon with 72 μL of AMPure on a 96 well plate. After 5 min at room temperature, beads were separated and washed twice with 80% ethanol and eluted in 30 μL of water. After purification samples were quantified by spectrophotometry (Nanorop, Roche, Mississauga, Canada) and normalized to a final concentration of 2 nM. Sequencing of the library pool was performed at the University of Guelph’s Advanced Analysis Centre using an Illumina MiSeq (San Diego, USA) and 2×250 chemistry.

### Microbiota assessment

The mothur package of algorithms (v1.32) was used for analysis [12]. Paired end reads were aligned. Sequences >244 bp or <237 bp in length, and those containing any ambiguous base calls or long runs (>8 bp) of holopolymers were removed, as were sequences that did not align with the correct region. Chimeras were detected using uchime [13] through mothur v1.31 rather than v1.32 because of a bug that was present in the mothur v1.32 at the time of analysis. Sequences from chloroplasts, mitochondria, Archaea and Eukaryotes were also removed. CatchAll was used to assess species richness [14].

Sequences were binned into operational taxon units (OTUs) at a 3% (0.03) dissimilarity level, and taxonomy was assigned using the Silva 16S rRNA reference database (http://www.arb-silva.de) [15]. Student’s t-test or ANOVA with Tukey’s post hoc test were used for comparison of continuous data, with a P value of <0.05 considered significant.

Subsampling was then performed to normalize sequence number for subsequent analyses. Coverage was assessed using Good’s coverage. Population diversity was described using the inverse Simpson’s index. Dissimilarity between the three groups was assessed through creation of dendrograms using the Yue & Clayton measure of dissimilarity (a measure of community structure, which considers shared OTUs and their relative abundances) and traditional Jaccard index (a measure of community membership, which considers the number of shared OTUs, not their abundance). Figures were generated by FigTree v1.4.0 (http://tree.bio.ed.ac.uk/). Parsimony and unweighted unifrac [16] tests were applied to evaluate the impact of feeding type and MRSA status on microbial population structure. Principal component analysis (PCA) was also performed. Analysis of molecular variance (AMOVA), a non-parametric evaluation of genetic diversity, was also applied to compare groups. Comparisons between MRSA positive and MRSA negative pigs were restricted to pigs from the same liquid-feeding farm to avoid potential bias. Significant (*P* < 0.05) indicator OTUs were identified. The core microbiota was assessed through identification of OTUs present in all samples at a minimum relative abundance of 1%.

### Availability of supporting data

The data set supporting the results of this article are available at the MG-RAST metagenomics analysis server (project 8129, http://metagenomics.anl.gov).

## Abbreviations

AMOVA: Analysis of molecular variance; ANOVA: Analysis of variance; MRSA: Methicillin-resistant *Staphylococcus aureus*; OTU: Operational taxon unit.

## Competing interests

The authors declare that they have no competing interests.

## Authors’ contributions

JSW and RF designed the study. MS provided input with study design, coordinated sampling and performed MRSA culture. MJ performed molecular studies. JSW performed data analysis and drafted the manuscript. All authors read and approved the final manuscript.
